# The first petrographic characterisation of a prehistoric rock crystal mine in the Swiss Alps

**DOI:** 10.1038/s41598-023-48914-8

**Published:** 2023-12-29

**Authors:** Thomas Hess, Josef Mullis, Leander Franz

**Affiliations:** 1 Department of Early Prehistory and Quaternary Ecology, University of Tübingen, Schloss Hohentübingen, 72070 Tübingen, Germany; 2 Department of Environmental Sciences, University of Basel, Bernoullistrasse 30/32, CH-4056 Basel, Switzerland

**Keywords:** Mineralogy, Petrology, Raman spectroscopy, Characterization and analytical techniques, Archaeology

## Abstract

Over the past decades, there has been increasing evidence for the prehistoric use of rock crystal in mountainous environments, including craft specialisation and long-distance exchange. Yet there are only a few known sites where the mineral was quarried in sustainable quantities. One of them is situated near Fiescheralp in the Upper Valais (Switzerland) and dates to the Early Mesolithic and a final stage of the Neolithic. Here we present the first petrographic characterisation of a prehistoric rock crystal mine in the Swiss Alps, involving a combination of different methods. The article provides a detailed description of the fluid inclusions within the quartz crystals and an overview over the related mineral paragenesis. This gives interesting new insights into the formation of the analysed fissure and allows comparing rock crystal artefacts found in other archaeological sites to this particular source. The results form the basis for further investigations concerning the circulation and distribution of the raw material in the past.

## Introduction

### Topographic and geological situation

**Figure 1:**
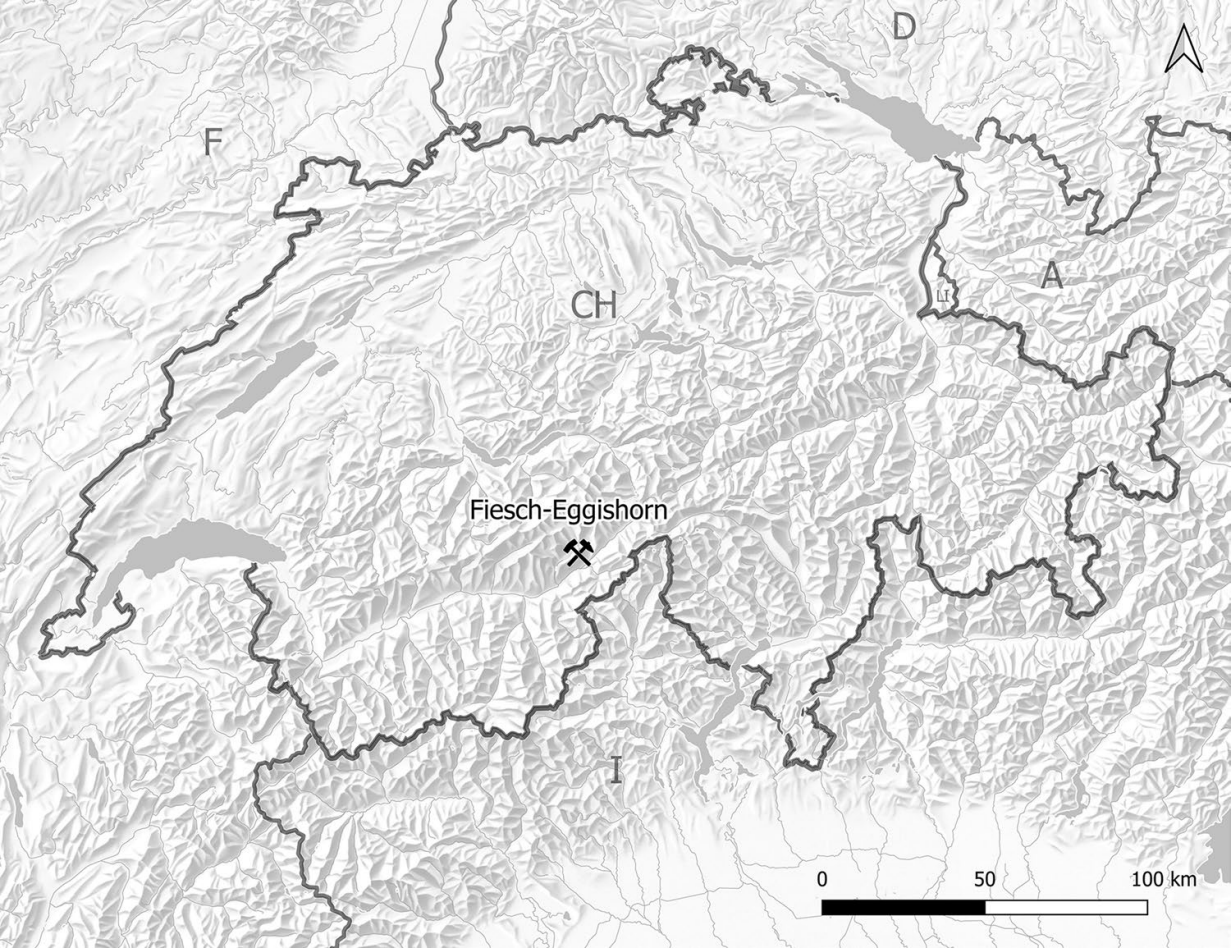
Location of Fiesch-Eggishorn. (created with QGIS, Version 3.10.5, https://www.qgis.org, source: Swisstopo, figure: T. Hess).

The archaeological site Fiesch-Eggishorn is situated in the Upper Valais near Fiescheralp at an elevation of 2575 m above sea level^1^ (Fig. 1). The terrain forms several small plateaus on a slope leading to a narrow passage that links the area with the valley of the famous Aletsch glacier. A steep mountain chain, running from the southwest to the northeast, protects the area from wind. The local flora is generally characterised by alpine meadows, while higher zones on the hillside are more scarcely vegetated. To the north, the Eggishorn mountain raises up to an elevation of 2927 m above sea level. Below the site—that is nowadays situated in close proximity to a hiking trail—there is a small artificial lake.

**Figure 2:**
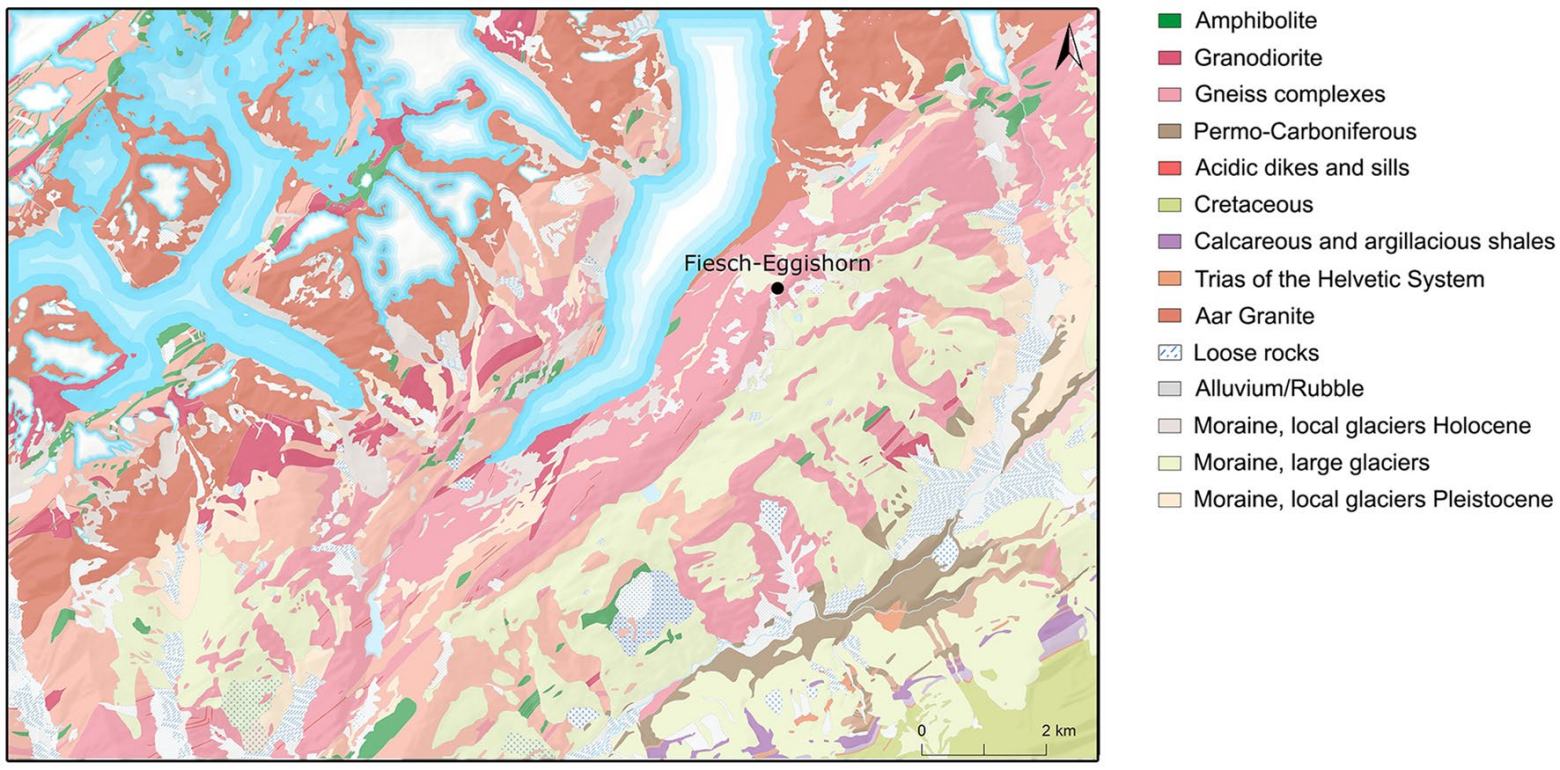
Geological map of the area. (created with ArcMap 10.8.1, https://desktop.arcgis.com, source: Swisstopo, figure: T. Hess).

The area is part of a migmatitic contact zone within the Aar Massif (which tectonically belongs to the Helvetic System), running parallel to the rock face described above^2,3^ (see Fig. 2). On the one hand, it is characterised by igneous rocks, such as granite, and, on the other hand, by metamorphic rocks, including gneiss and amphibolite. This becomes also evident by the fact that a nearby rock glacier contains both materials. While the younger metamorphic rocks have formed in the course of the Alpine orogeny during the Tertiary, the crystalline basement is considerably older in age and mainly dates to the Palaeozoic (Variscan orogeny, ~ 300 Mio. years ago)^4^. In addition, outcrops of rocks that are of pre-Variscan origin were documented. The Permo-Carboniferous rocks in the Rhone Valley mainly consist of Verrucano, the Trias of the Helvetic System includes evaporites, sandstones and phyllites. The Cretaceous rocks further to the south are already part of the Penninic System and comprise of limestones and schists.

Generally, the area is strongly influenced by glacial processes and the sediment is composed of alternating layers of fine-grained clay and coarser sand, representing several melting events and ice advances. The surrounding bedrock consists mainly of biotite gneiss. The site—that is almost year-round covered by snow and ice—offers a great view over the Rhone Valley and several peaks of the Pennine and Lepontine Alps. Among others, the entrance to the Binn Valley, known for its rich and diverse mineral deposits, the Simplon Pass, linking Switzerland with Northern Italy, and the passage to the Mesolithic open-air site Alpe Veglia (I)^5^ are visible. Depending on the sight, it is even possible to detect the famous Matterhorn further to the southwest. In close vicinity of the site, several large boulders that could have served as campsites and quartz-bearing fissures were documented. During the construction of a cable car leading to the summit of Eggishorn mountain, a large quartz crystal group with a weight of several kilos was discovered.

### Research history

In the 1990’s, the amateur archaeologist Gertrud de Vries (1922–2011) discovered several peculiar objects made of rock crystal near a construction site within the skiing area above Fiescheralp. De Vries, who owned a holiday apartment in Valais, was working as a volunteer for the Canton of Basel and after she has passed away, the finds were brought to the archive of the Cantonal Archaeological Service. In 2011 the material was handed over to the main author who conducted surveys in the area during the following summer and was able to relocate the site and identify it as a prehistoric rock crystal mine. In the framework of regular inspections during the next years and a field campaign in 2019, the archaeological context was reconstructed. Furthermore, the lithic assemblage was systematically analysed and the finds were drawn and documented photographically. Typo-technological analyses allowed to determine the represented time periods and made it possible to link the place with other archaeological sites in the surrounding area^1^.

Between 2021 and 2022, a geochemical characterisation of the raw material was conducted at the Department of Environmental Sciences of the University of Basel. It is the first detailed description of a prehistoric rock crystal procurement site, involving different petrological methods. The study of fluid inclusions in the context of archaeological research has already yielded interesting results in the Canton of Grisons^6^, southeastern France and northwestern Italy^7–9^, and the Tyrolean Alps^10^. Other projects involved the application of Raman spectroscopy for the analysis of prehistoric rock crystal in the Czech Republic and Poland^11,12^.

### Archaeological and geological features

**Figure 3:**
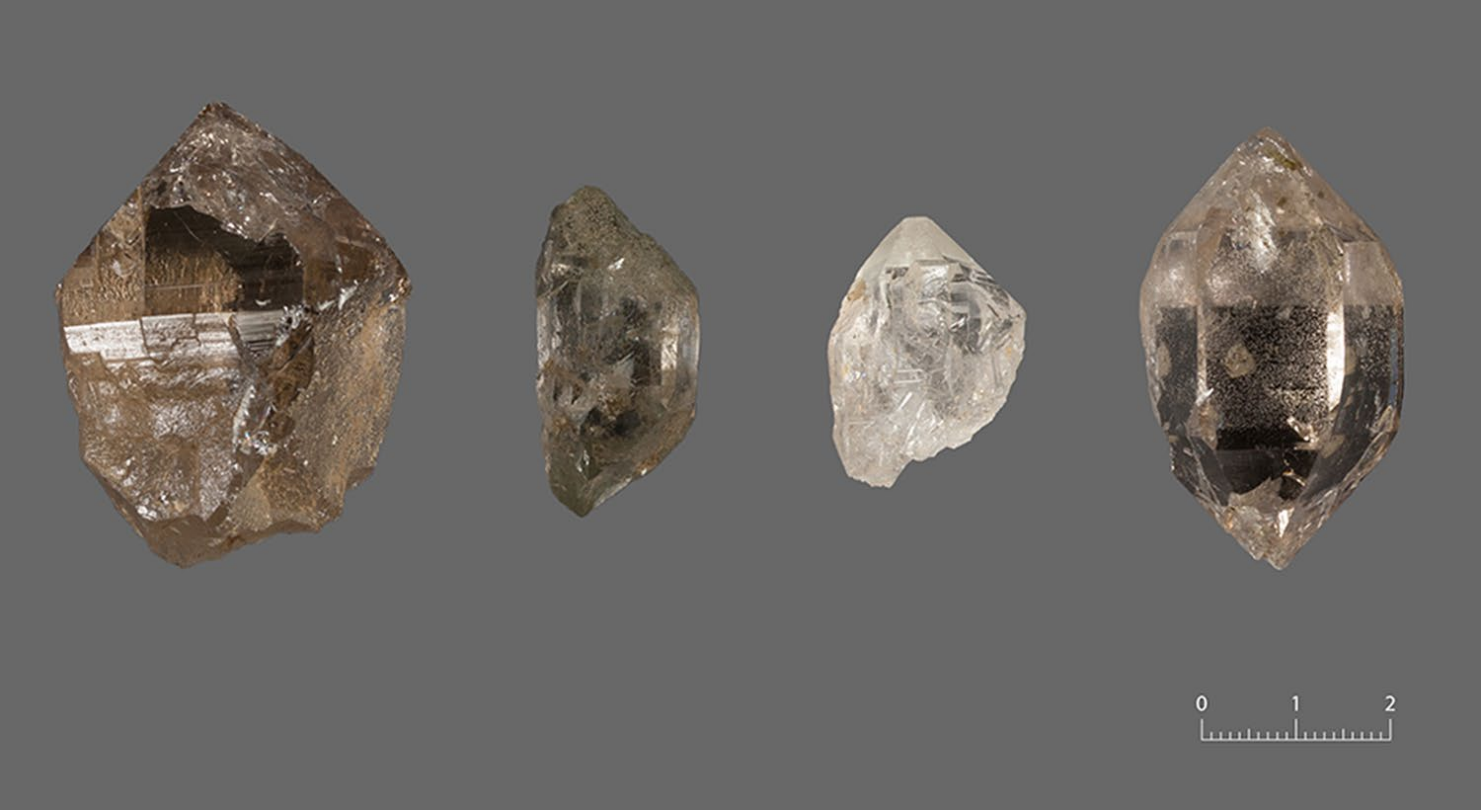
Photographs of different samples from Fiesch-Eggishorn (figure: T. Hess).

As a consequence of constructions on a skiing-slope, the uppermost archaeological layers were partly eroded and within an area of about 20 m^2^ hundreds of objects consisting of rock crystal were visible on the surface. Among them are several pieces with clear signs of lithic artefacts (as opposed to objects created by natural processes), such as bulbs of percussion, platform remains, and Wallner lines (concentric lines or ridges indicating the direction of the applied force that are interpreted as a marker of intentional flaking). In the framework of a field campaign in 2019, a trial trench with a length of 2 m and a width of 1 m was opened. Parts of the stratigraphy were still intact and a few diagnostic finds were encountered in situ. Furthermore, in the southwestern corner of the trench, it was possible to document the original surface of the fissure where the raw material was quarried. It runs perpendicular to the foliation of the host rock (a K-feldspar biotite orthogneiss) and includes a quartz vein as well as the remains of removed crystals. Due to weathering processes, the surrounding rock is strongly leached and the area is filled with coarse chlorite and biotite sand. Nowadays, the quartz prisms are only loosely attached to the rock surface. Generally, the crystals are characterised by an extraordinary transparency. Some pieces show a slightly darker colour (smoky quartz) or are stained by iron oxides. Besides the normal prismatic habit, double-ended crystals were documented (see Fig. 3). Occasionally, there are specimens with steep rhombohedral faces (so-called Tessin habit or Penninic habit) among the archaeological artefacts. These must reflect imports from an area further to the south (e.g. the Binn Valley).

The analysed assemblage consisted of 1134 pieces and had a weight of 2572 g^1^. In the framework of typo-technological analyses, the objects were systematically investigated and drawings of the finds were created. Additionally, each piece was studied using a stereomicroscope. Besides artefacts that are typical for an Early Mesolithic age, such as microliths and microburins, large blades and bifacially retouched tools, pointing to a younger stage of the Neolithic, were documented. It was possible to link the procurement site with several Mesolithic localities in the surrounding area that yielded radiocarbon dates between 8000 and 6500 BC^13^. According to current knowledge, it is the oldest site of this kind in the entire Western Alps. This makes it a significant archaeological discovery for the study of the use of resources in the past. As the Rhone Valley leads to a warm and dry climate, it is possible that the locality was already accessible at a relatively early stage of the Holocene. Additionally, a recent radiocarbon date proves the presence of people at Fiescheralp during the Early Bronze Age, as part of a system of vertical transhumance cf.^6,14–17^. This links the site with the rock crystal workshop at Hospental-Rossplatten (UR), dating to the Copper and Bronze Age^18,19^.

Until today, there are only two other rock crystal mines in Europe, dating as far back as the Mesolithic. One of them is the site Riepenkar in the Tyrolean Alps at an elevation of 2800 m above sea level^20^. The archaeological features display great similarities to Fiesch-Eggishorn. In addition to an Early Mesolithic occupation, the outcrop was also used during a younger phase of the Neolithic. Another rock crystal procurement site was discovered at Fuorcla da Strem close to a glacier at the border between the Cantons of Uri and Grisons in the eastern part of Switzerland^21,22^. A radiocarbon date places the age of the finds to the 6th millennium BC.

## Materials & methods

The samples for petrographic and mineralogical analyses comprised of unworked rock crystals—in some cases containing remains of the bedrock—that formed within the described fissure. Those were compared to each other and studied with different empirical methods to understand the tectonic and hydrothermal processes leading to their formation. With its fluid and solid inclusions, quartz crystal records the history of the alpine-type fissures and their mineral parageneses^23,24^. In Switzerland, it is currently possible to distinguish between four different zones, running from the northwest to the southeast, depending on the composition of the fluid^23,25–29^. Those in turn are linked with the host rock of the respective fissures and the occurrence of specific minerals.

Similar as in case of important sources of flint, chert or obsidian^30–33^, a petrographic description of the material in question involving a variety of different methods (including analyses that require the destruction of a sample) opens the possibility to conduct provenance analyses of archaeological objects even without necessarily having to destroy them.

### Microscopy

**Figure 4:**
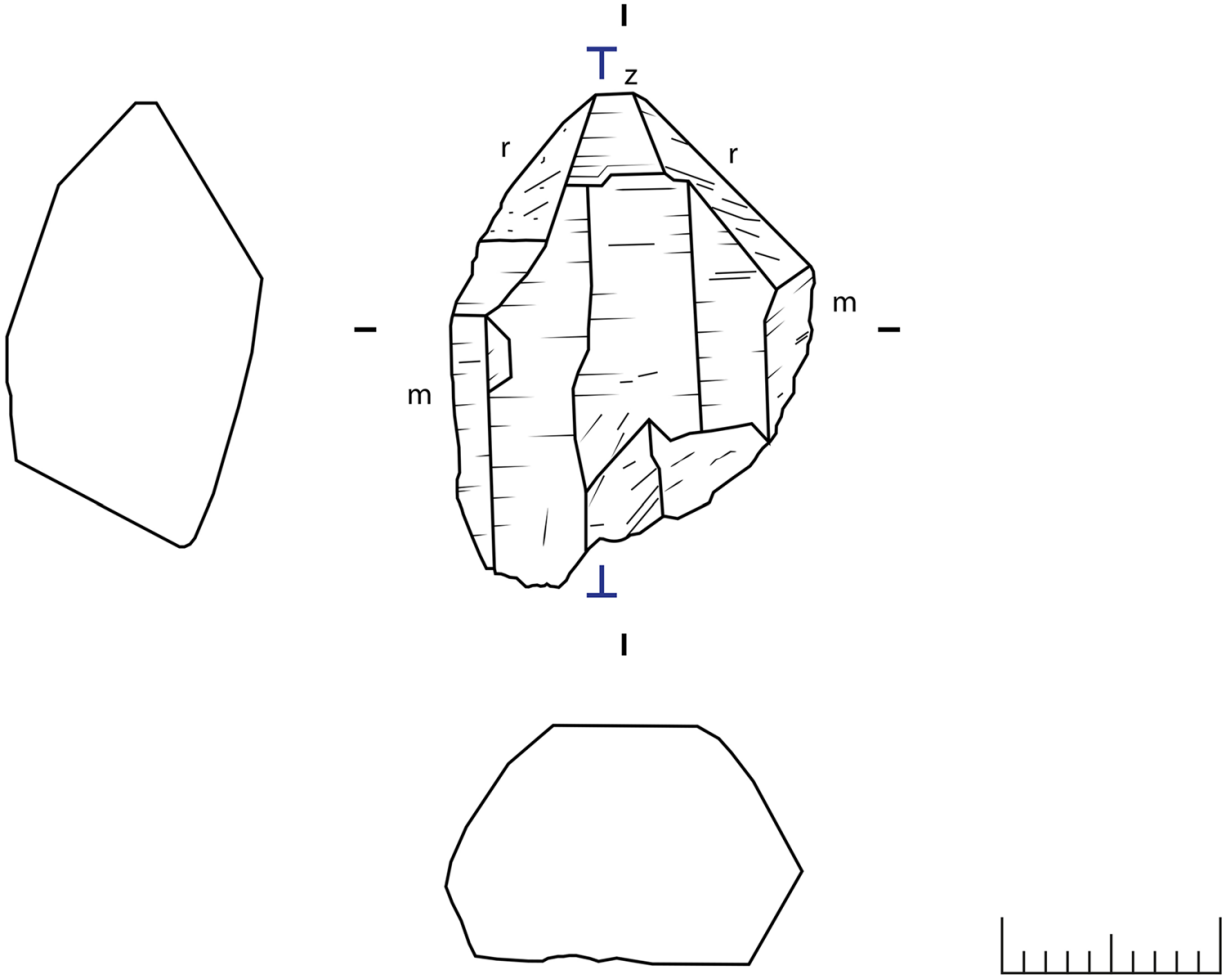
Drawing of the sample used for the analysis with the position of the thin-section (drawing: T. Hess).

The morphology of several quartz crystals collected in the investigated fissure from Eggishorn was characterised by optical microscopy. A comparison between unworked samples and archaeological artefacts discovered at the site allowed the identification of repeatedly occurring types of fluid inclusion assemblages. Minerals that were incorporated as solid inclusions within the quartz or precipitated after quartz growth were documented descriptively, in order to reconstruct the mineral paragenesis. In case of a representative piece, a thin-section with a thickness of 500 µm (a so called thick-section) was produced in order to study it in more detail and conduct a series of measurements using microthermometry (Figs. 3, 4). The original sample had a length of 28.2 mm, a maximum width of 19.8 mm and a weight of 9.0 g.

### Microthermometry

A doubly polished thick-section was investigated using microthermometry on two different freezing and heating stages from Chaixmeca^34^ and Linkam Inc THMSG 600, respectively. Calibration was performed with synthetic fluid inclusion standards in a temperature range between − 56.6 and 374.1 °C and verified with appropriate chemicals from Merck Corporation. Measurement precision between − 30 and + 30 °C is 0.1 °C and outside this range up to 1 °C.

### Volume fraction estimation

Volume fraction estimation of liquid and vapour phases of fluid inclusions were estimated by comparing the images to samples evaluated by the spindle-stage technique at the University of Bern (cf.^35^).

### Composition of the fluid inclusions

In presence of CO_2_ vapour (within the bubble) and CO_2_-clathrate, the salinity and the CO_2_ concentration in fluid inclusions are derived from the melting temperatures of ice and CO_2_-clathrate (^36^; CLATHRATES, Program 3: ICE, version 12/2) (Supplementary Table 1, population 1 and 2). In inclusion populations where no CO_2_ vapour and clathrate could be observed, salinity is derived as NaCl equivalents from the ice melting temperature according to Potter et al.^37^, Bodnar^38^ and Bakker^36^ (FLUIDS, Program 1: BULK, version 01/03) (Supplementary Table 1, population 3 and 4). The salinity is given as NaCl equivalents, as the measured eutectic temperatures of the fluid system (CaCl_2_ × 6H_2_O) and the melting temperatures of hydrohalite (NaCl x 2H_2_O) in Supplementary Table 1 refer to a CaCl_2_/NaCl-ratio between 0.07 and 0.15^39^.

### Raman spectroscopy

Minerals and fluid inclusions within the quartz crystals and mineral fragments from the host rock were identified by confocal Raman spectroscopy using a Bruker Senterra dispersive microscope spectrometer. Translucent mineral phases and fluid inclusions were analysed with a green solid-state Nd-YAG laser at 532 nm with 20 mW, and with a red direct diode laser at 785 nm with 100 mW and measuring times of 10–100 s. For opaque phases, a red direct diode laser at 785 nm with 10–25 mW and measuring times of 10–30 s was utilized. Measurements were performed using an objective lens with a magnification of 50 × and an aperture of 50 μm. Mineral identification was accomplished with the RRUFF database^40^ while liquid and gaseous components were identified using the data of Schrötter & Klöckner^41^.

## Results

### Mineralogical characterisation

The mineralogical characterisation is based on a detailed petrographic description, including optical microscopy, the creation of drawings of the studied samples, and the geochemical analysis of the minerals using Raman spectroscopy.

Natural quartz samples from the archaeological site near Eggishorn are of prismatic habit by elongation along the c-axis, with the hexagonal prism *m* in combination with the trigonal rhombohedra *r* and *z* as dominant forms. Much less frequent are trigonal pyramids. Sutures running parallel to the crystallographic c-axes upon the prismatic and rhombohedral faces as well as striations perpendicular to the crystallographic axes are common.

**Figure 5:**
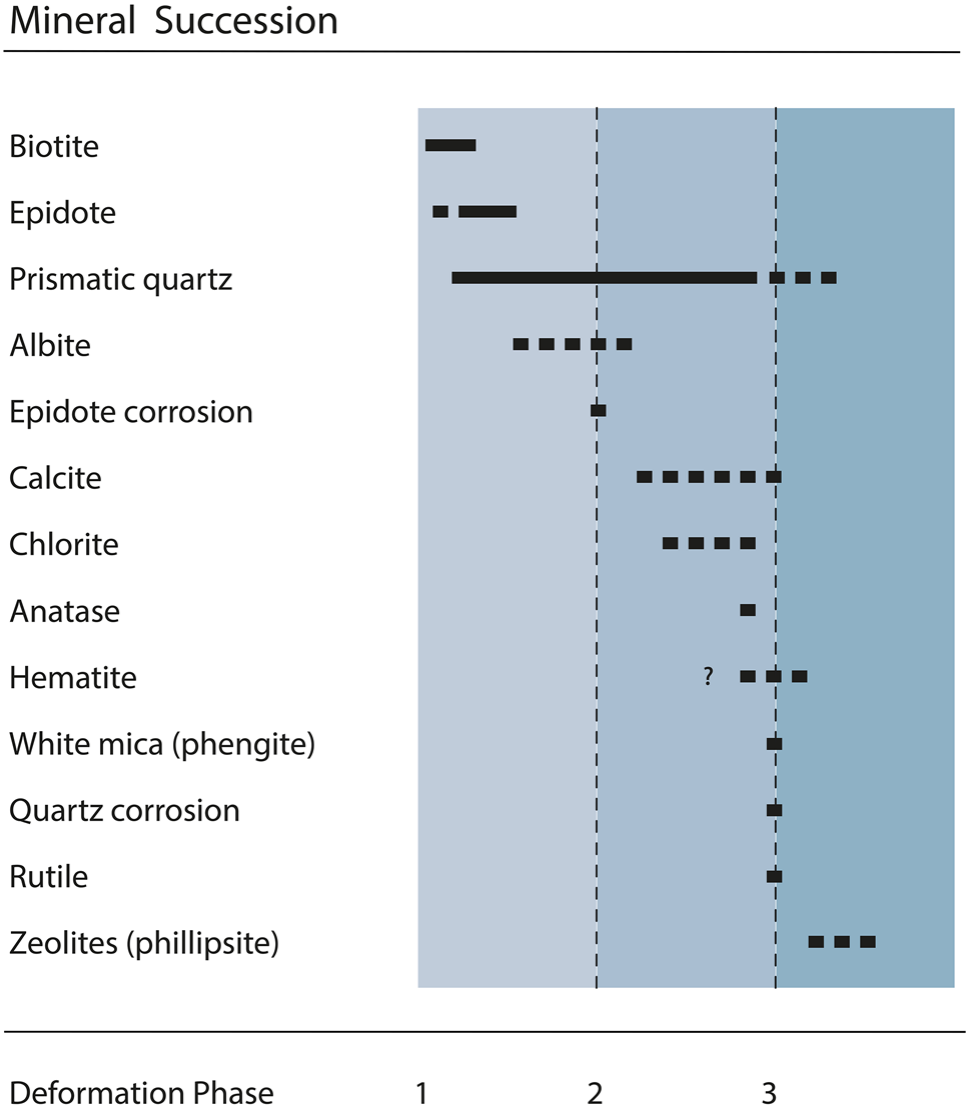
Mineral succession observed in the sample (figure: J. Mullis).

The entire fissure minerals that crystallised before, during and after quartz growth are displayed as relative mineral succession in Fig. 5. Prismatic quartz with a macromosaic structure^42^ is the dominant fissure mineral. Small relicts of minerals crystallising before and during quartz growth are incorporated as solid inclusions in quartz. Together with the minerals that precipitated after quartz growth, their relative succession can be established and subdivided into an early, intermediate, and late section of the mineral paragenesis (see Fig. 5).

After fissure opening (first deformation phase), biotite, epidote, and prismatic quartz began to crystallise. Together with albite, they represent the early mineral paragenesis. After the second deformation phase, epidote corrosion occurred while calcite, chlorite, anatase, and hematite crystallised. In the course of a third deformation phase, white mica (phengite) was formed. This process was succeeded by minor quartz corrosion. The abundance of phengite, hematite, rutile, and the zeolite mineral subgroup phillipsite define the late stage of the mineral paragenesis.

### Fluid inclusions

**Figure 6:**
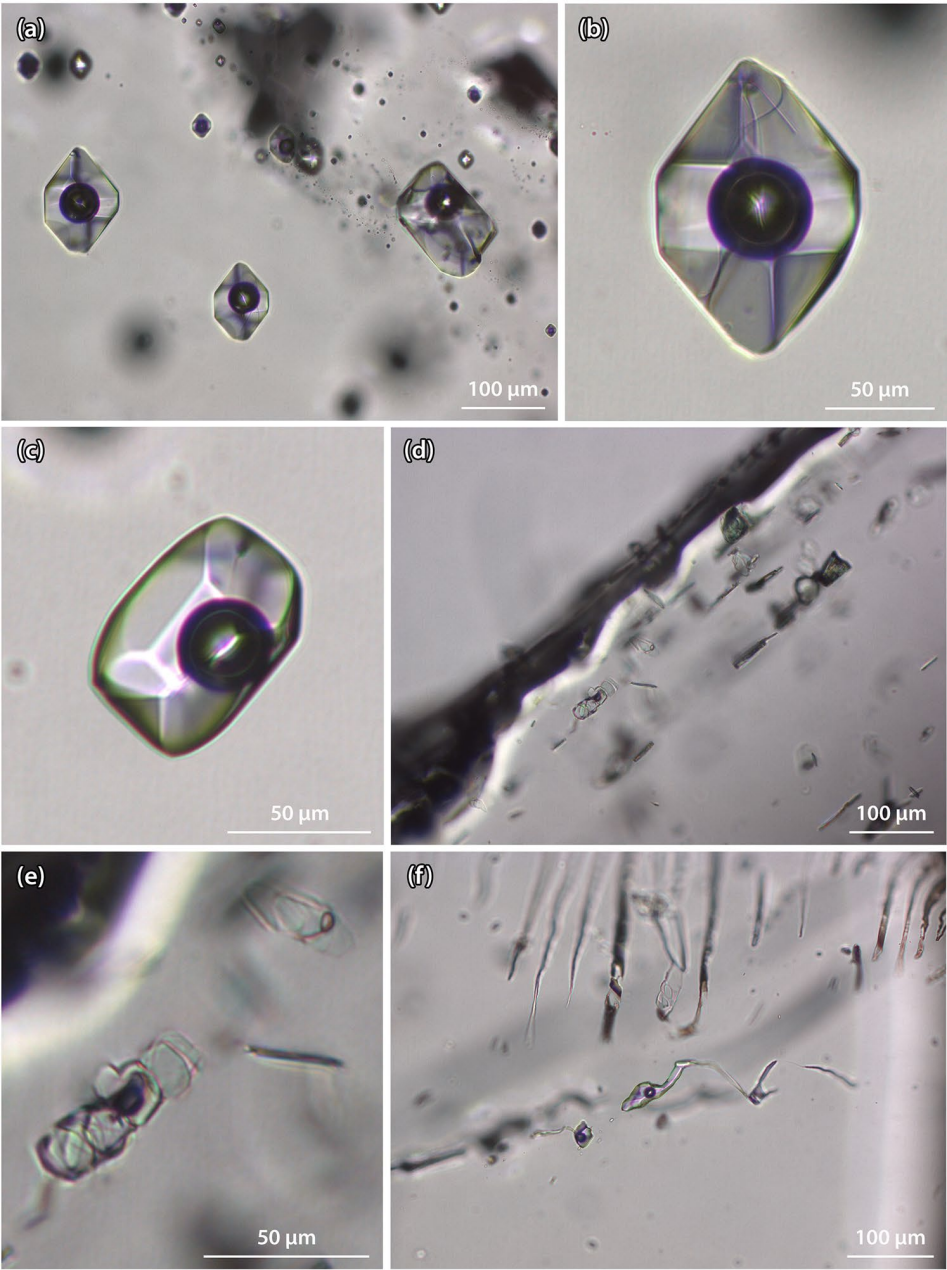
Fluid inclusions observed within the sample. (**a**) Large pseudosecondary fluid inclusions of the first population. (**b**) Fluid inclusion of the first population with rutile whisker. (**c**) Secondary fluid inclusion of the second population. (**d**), (**e**) Chlorite-rich inclusions of the third population, appearing in layers. (**f**) Elongated secondary fluid inclusions of the fourth population. (photographs: J. Mullis, figure: T. Hess).

Four populations of water bearing fluid inclusions were discriminated: 1. Large pseudosecondary fluid inclusions with a size between 50 and 150 µm that formed during the first part of quartz growth (see Fig. 6a,b). 2. Large secondary fluid inclusions within the second growth stage of the quartz crystal (see Fig. 6c). 3. Water bearing fluid inclusions in population 3 that formed simultaneously with chlorite that fell as already formed mineral from the fissure wall on the upward looking quartz faces (phantom growth) (see Fig. 6d,e). 4. Large (up to 300 µm), elongated secondary fluid inclusions, cutting the quartz crystal (see Fig. 6f). All fluid inclusion populations are filled with an aqueous saline solution and contain a vapour bubble with a size between 8 and 12 vol%. Microthermometric analyses indicate the presence of a very small amount of CO_2_ dissolved in aqueous solution of inclusion population 1 and 2. This has been confirmed by Raman spectroscopy (see Fig. 8). Rutile and small, not further specified, anisotropic minerals are present as daughter minerals inside the fluid inclusions. In population 3, chlorite is the dominant mineral. Because of the reduced transparency, only a small number of fluid inclusions could be measured.

**Figure 7:**
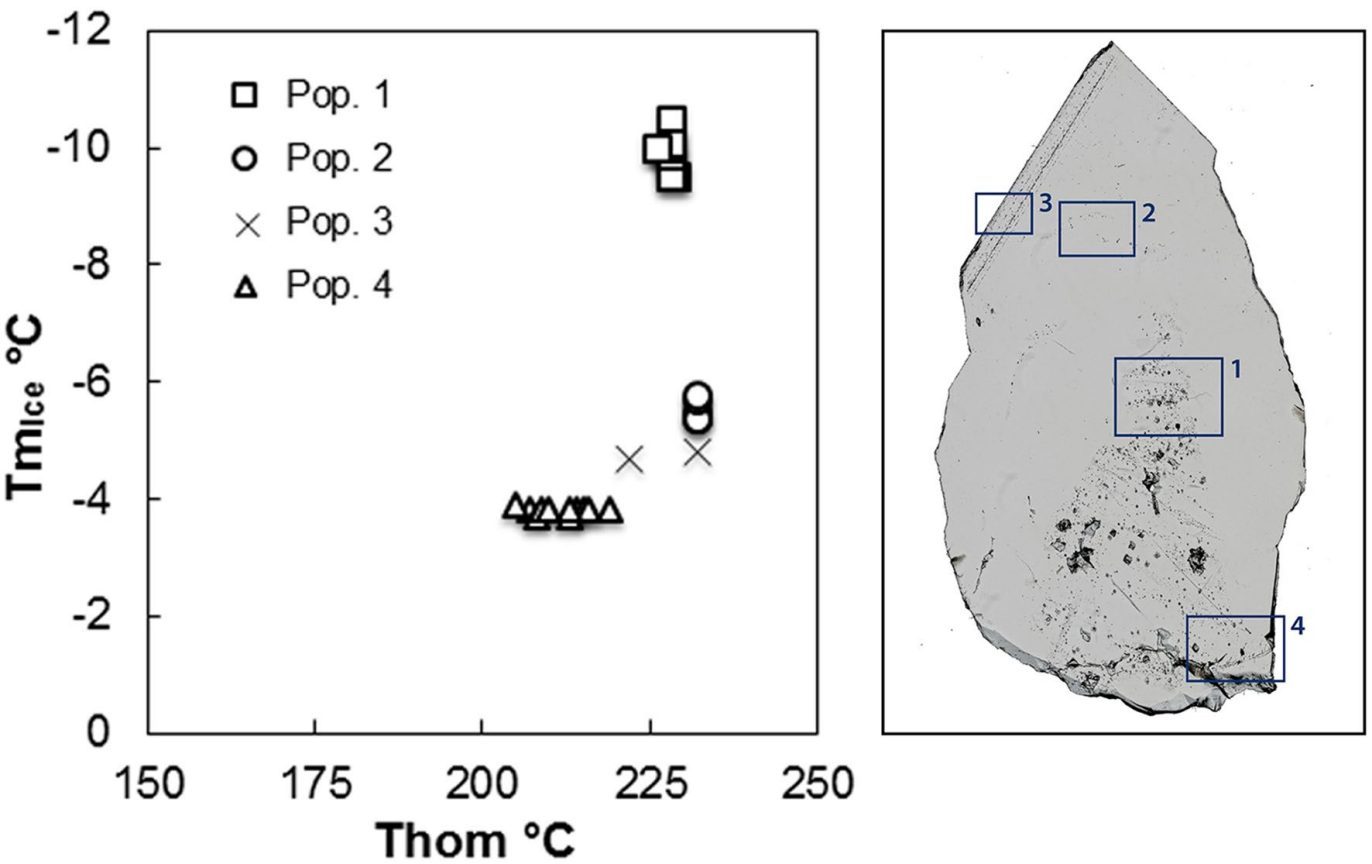
Left: Plot showing the melting temperature of ice versus the homogenisation temperature of different fluid inclusion populations (figure: J. Mullis). Right: Thin-section of the sample with different fluid inclusion populations, indicating the studied sections. (photograph: T. Kipfer, figure: T. Hess).

The mean values of ice melting temperatures of all inclusion populations range from − 9.9 to − 3.8 °C, and those of total homogenisation temperatures are situated between 232 °C and 211 °C (Fig. 7 and Supplementary Table 1). Salt dissolved in the aqueous solutions decreases from the first to the fourth inclusion population from 3.5 to 2.0 mol% NaCl equivalents. CO_2_ decreases from 3.2 mol% in the earliest to ≤ 1 mol% in the youngest fluid inclusion population (see Supplementary Table 1). This trend is suddenly interrupted by the composition of inclusion population 2, showing the smallest salinity (1.4 mol% NaCl equivalents) and the highest CO_2_ content (3.7 mol%). Based on the remarkable amount of rutile whiskers included as daughter minerals within fluid inclusions (see Fig. 6b), it is possible to state that the aqueous solution of populations 1 and 2 must have been enriched with dissolved titanium during fluid inclusion formation.

## Discussion and conclusion

Alpine fissures situated in various geological, tectonic, and metamorphic environments result in different mineral parageneses^23,25–29^. Therefore, the detailed description of a fissure system with its characteristic mineral succession and the report of growth parameters allow determining the origin of almost every quartz sample in the Alps. This is of fundamental interest for archaeological research.

The presented study demonstrates various tectonic events that opened and deformed the Alpine fissure system. The chemical composition of the aqueous fluids changed systematically as a consequence of tectonic deformation and decreasing temperatures under retrograde metamorphic conditions, altering pre-existing rocks such as biotite gneiss and amphibolites. According to the interplay of various physical and chemical factors, the fluid-rock equilibrium was repeatedly disturbed and led to the precipitation of the mineral succession documented at the site near Fiescheralp.

### Formation of the fissure

**Figure 8:**
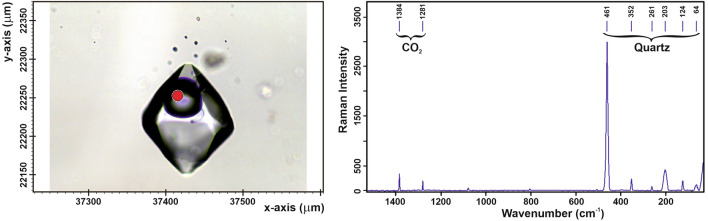
Left: Photograph of a pseudosecondary, low salinity aqueous fluid inclusion of the first generation with a CO_2_-bearing bubble. Notice the bent rutile whisker below the bubble. Right: Raman spectrum with the bands of quartz and CO_2_ (figure: L. Franz).

Initial opening of the fissure and repeated deformation events led to fluid-rock-disequilibria resulting in mineral dissolution and precipitation cf.^23,25–29^. As temperature, pressure and fluid composition evolved during retrograde metamorphism, three mineral parageneses formed (see Fig. 5). The first two fluid inclusion populations (CO_2_ > 3 mol%) (see Supplementary Table 1) refer to low and medium grade greenschist-facies metamorphic conditions^25,28,29^. A high amount of dissolved titanium triggered the growth of large rutile whiskers within fluid inclusion population 1 (see Fig. 8). In addition, biotite, epidote, prismatic quartz, and albite crystallised from this early fluid. The second fluid inclusion population shows a considerable decrease of salt and increase of CO_2_ compared to the first population. Such a sudden change in fluid composition refers to a temporary fluid advection due to an additional deformation event of the fissure. As a consequence, epidote corrosion and simultaneous calcite precipitation occurred. Fluid inclusions of population 3 formed simultaneously with chlorite that accidentally fell as already formed mineral from the fissure wall to the quartz surface (phantom incorporation). The mentioned features reflect a characteristic chemical reaction pattern. The end of chlorite precipitation and the appearance of a very small amount of white mica towards the end of quartz growth are interpreted as a consequence of a small advection of dissolved, but non-detected CO_2._ This is linked with an additional fissure deformation event^23,26,28^. Finally, quartz crystals characterized by a prismatic habit precipitated in a water-rich fluid with less than 10 mol% CO_2_ under low to medium grade greenschist-facies metamorphic conditions^25,28,29^.

### Archaeological implications

The presented work is of high significance in order to petrographically characterise the investigated fissure. A detailed description of the mineral succession and the composition of the fluid inclusions with a combined methodological approach, allows unequivocally identifying the rock crystal in question. It forms the basis for further analyses, aimed at comparing finds from other archaeological sites of similar age to the raw material source on Fiescheralp. This goal could either be achieved by studying a selected piece (such as a preparation flake), in thin-section^6,7^, or by the optical identification of the same mineral paragenesis and the characteristics of fluid inclusions^29^. Of particular importance in this context is the Mesolithic open-air site Alpe Veglia in Northern Italy, only 16 km to the south as the crow flies, or the rock shelter site Alp Hermettji on the foothills of the Matterhorn^43,44^. A petrographic comparison of lithic artefacts discovered at the former site with rock crystal deposits in the study area has already yielded interesting preliminary results. Based on the crystal habit and the fluid inclusions, it was possible to state that the respective groups used raw material from the Aar Massif (H_2_O zone) as well as tectonic units belonging to the Penninic system within a fluid zone containing ≥ 10 mol% CO_2_ (CO_2_ zone). In additon, Cretaceous flint and radiolarian chert occurring in the proximity of large lakes near Como and Varese was imported over a distance of more than 80 km. The mentioned provenance analyses allow the reconstruction of Mesolithic transit routes and mobility patterns in Alpine environments. Particularly interesting is the fact that there is a seasonal component concerning the accessibility and procurement of rock crystal, as the mining sites are situated in high altitude zones. However, recent investigations have come to the conclusion that prehistoric groups inhabiting mountainous regions have specialised in the production of artefacts made of crystalline quartz^1,15^, and some authors have pointed out the possibility of an intra-alpine exchange network that facilitated the circulation of the precious raw material during the Mesolithic^45^. Similarly, it is important to test the hypothesis that the rock crystal for the production of Neolithic artefacts found at the megalithic burial site Sion-Petit-Chasseur^46^ has its origin in the Upper Valais. Furthermore, objects made of rock crystal (including unworked prisms that could have played a role in the framework of rituals) were found in Neolithic lakeside settlements. While in case of greenstone^47^, flint and chert^33^, as well as marine shells^48^ potential routes and trade models have been studied in great detail, much of the acquisition and distribution of rock crystal remains unknown. By analysing the provenance of rock crystal artefacts, it might be possible to get insights into social relations and cultural boundaries throughout time. Studies on land-use patterns of hunter-gatherers^49^ suggest that geological and geomorphological factors play a key role for the understanding of settlement strategies and the way in which prehistoric people interacted with their environment (cf.^50,51^). By following particular geological formations—leading to the abundance of raw material outcrops, potential campsites and other important resources—people were able to adapt faster to previously unknown landscapes^52^. Therefore, an in-depth study and a comparison of topographic parameters allow for the creation of GIS-based models aimed at detecting new localities and connections between raw material sources and known archaeological sites. In the framework of an ongoing project, the authors are investigating this topic by applying a combined approach including non-destructive methods, such as the identification of the crystal habit (e.g. prismatic habit, Penninic habit, or sceptre like quartz)^53,54^, a description of the mineral succession, and an estimation of the composition of fluid inclusions (hydrocarbons, CH_4_, NaCl, H_2_O or CO_2_)^25,28^ in order to determine the provenance of lithic artefacts made of rock crystal on a regional level. The exact localisation of a specific fissure system that was used in prehistoric times requires additional methods (involving the destruction of a small portion of a sample), such as microthermometry, Raman spectroscopy, cathodoluminescence and LA-ICP-MS^12,55,56^.

### Supplementary Information

Supplementary Table 1.

## Data Availability

The data that support the findings of this study are included in the article. Further information (including the Raman spectra generated by L.F.) is available from the corresponding author [T.H.] upon reasonable request.
